# Supplementary material for: Role of CYP3A5 in Modulating Androgen Receptor Signaling and Its Relevance to African American Men with Prostate Cancer
Source: Cancers (Basel). 2020 Apr 17;12(4):989. doi: 10.3390/cancers12040989 (PMC7226359; doi:10.3390/cancers12040989)
Supplement: Supplementary file 1 [file cancers-12-00989-s001.pdf]

Supplementary Materials: Role of CYP3A5 in Modulating Androgen Receptor Signaling and Its Relevance to African American Men with Prostate Cancer

Priyatham Gorjala, Rick A. Kittles, Oscar B. Goodman Jr. and Ranjana Mitra

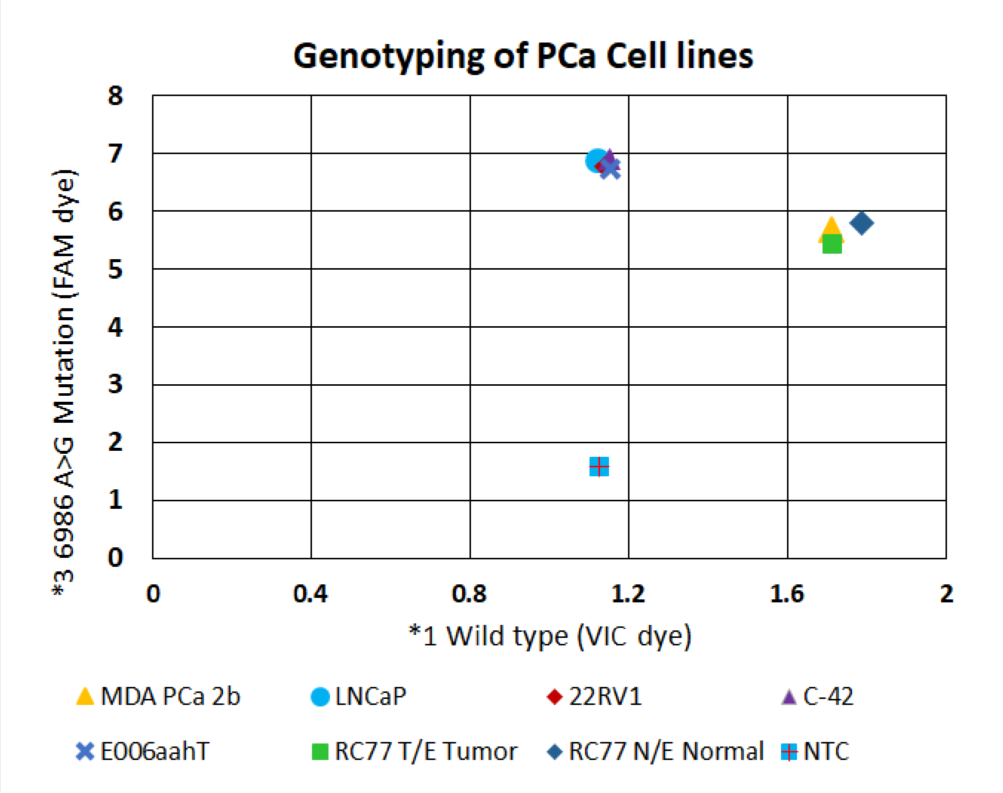

Figure S1. Genotyping of prostate cancer cell lines: Genomic DNA from the above mentioned cell lines were used to determine the presence of CYP3A5 wild type (*1) and mutant (*3) alleles. The x –axis represents the wild type allele, as the VIC dye is associated with the wild type detection probe. The y-axis represents the mutant (*3) allele as the FAM dye is associated with the mutant detection probe. The NTC is the no template control to set the base line. The LNCaP, 22RV1, C4-2, and E006aahT show only FAM signal (only *3) where as MDAPCa2b and RC77 T/E and N/E (derived from same patient) show presence of both FAM and VIC signals (*1/*3). 50 ng of DNA was used for the assay, the assay was repeated more than three times with different isolates from different passages an representative experiment is shown.

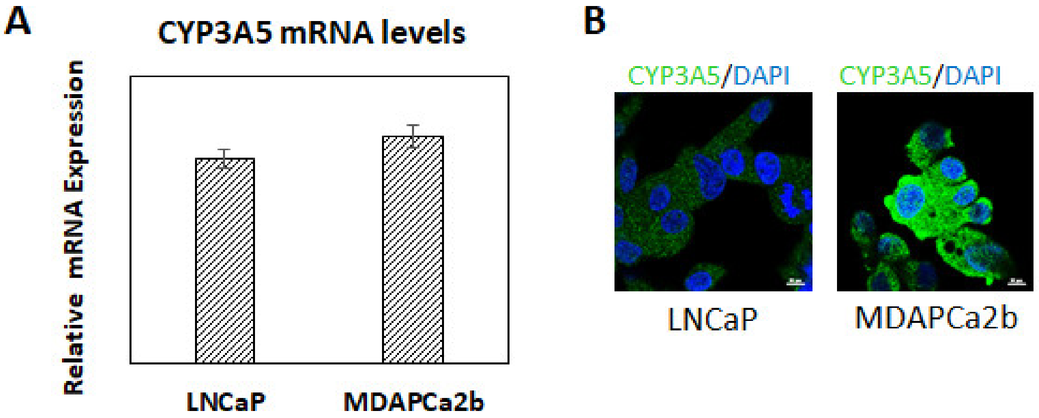

Figure S2. Comparison of CYP3A5 mRNA and protein expression in LNCaP and MDAPCa2b cell lines. (**A**) Equal amount of RNA was used to generate c-DNA from both the cell lines which was further used in qPCR experiment to determine CYP3A5 mRNA expression levels. GAPDH was used as a control to normalize. (**B**) Confocal microscopy was performed after staining the cells with CYP3A5 primary antibody and AF488 secondary antibody. The nucleus was stained with DAPI. The size bar is 10μm. A representative z-stack passing through the middle of the nucleus is shown. The pictures were taken at the same laser power to compare CYP3A5 expression levels.

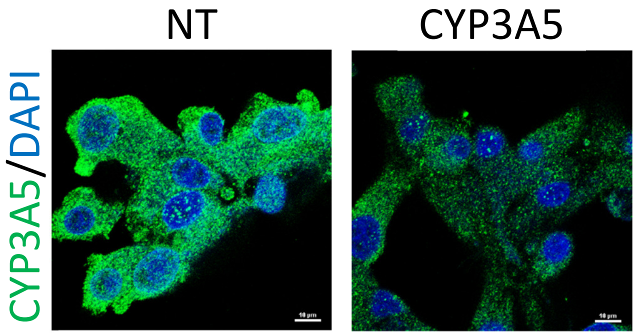

Figure S3. Effect of CYP3A5 siRNA on CYP3A5 protein levels. MDAPCa2b cells were transfected with NT (non-target) and CYP3A5 siRNA pool. On the 4th day after transfection the cells were fixed permeabilized and stained with CYP3A5 primary and AF488 secondary antibody. The nucleus was stained with DAPI (blue). The size bar is 10μm. A representative z-stack passing through the middle of the nucleus is shown. The pictures were taken at the same laser power to compare CYP3A5 expression levels.

Table S1. CYP3A5 genotyping analysis of prostate cancer cell lines.

	***1 Wild Type**	***3 6986 A>G Mutation**	
**Sample Name**	**Allele X Rn/Delta Rn**	**Allele Y Rn/Delta Rn**	**Call**
LNCaP	1.11414	6.97977	Allele2_MGB
22RV1	1.14396	6.8269	Allele2_MGB
C-42	1.1534	7.06453	Allele2_MGB
MDA PCa 2b	1.68542	5.71622	Both
E006aahT	1.14714	6.59419	Allele2_MGB

RC77 T/E Tumor	1.74107	5.30903	Both
RC77 N/E Normal	1.7183	5.75658	Both
NTC	1.12058	1.58675	Undetermined

NTC is the negative control (no target DNA) for setting the baseline for both the FAM and VIC labelled probes. Allele X (*1, wild type) gives values for the VIC signal which is bound to 5′ of the probe specific to wild type. The allele Y (*3, mutant) gives values for the mutant allele as the FAM is attached to 5′ of the mutant specific probe.

Table S2. Table showing fold change with P value in AR downstream-regulated genes.

Gene symbol	Fold Change	*p* Value	Gene Symbol	Fold Change	*p* Value
ABCC4	-1.4779	0.158456	ORM1	1.839	0.007383
ABHD2	-1.1285	0.828411	ORM2	2.0262	0.318749
ACSL3	-1.7475	0.031188	PAK1IP1	-3.254	0.015956
ADAMTS1	-1.468	0.067877	PGC	-1.3696	0.428019
ALDH1A3	-1.1699	0.657286	PIAS1	-1.3618	0.391551
APPBP2	-1.5388	0.153091	PIK3R3	-2.6605	0.017855
AR	-1.6888	0.06232	PMEPA1	-2.1143	0.030902
CAMKK2	-1.759	0.053974	PPAP2A	-1.3249	0.199678
CENPN	-2.1036	0.021776	RAB4A	-1.3636	0.387594
CITED2	-1.9274	0.041855	REL	-1.211	0.600392
ACKR3	2.6822	0.062786	RELA	-1.3016	0.431876
CYP2U1	-1.6126	0.122682	RHOU	-2.1077	0.042831
DBI	-1.2703	0.025292	SEC22C	-1.648	0.048733
DHCR24	-2.1823	0.036002	SGK1	1.373	0.322701
EAF2	1.1818	0.144587	SLC26A2	-1.5196	0.131967
ELK1	-2.9308	0.037614	SLC45A3	-4.5619	0.002423
ELL2	-3.251	0.003617	SMS	-1.3771	0.30469
ENDOD1	-1.3374	0.403678	SNAI2	3.32	0.00497
ERRFI1	-1.5812	0.377001	SORD	-1.5679	0.014492
FAM105A	-1.0251	0.941778	SP1	-1.9549	0.070468
FKBP5	-4.4312	0.001668	SPDEF	-2.5759	0.012049
FOS	-1.1142	0.897169	SRF	-3.0659	0.019875
FZD5	-2.9479	0.057798	STEAP4	-1.4217	0.663406
GUCY1A3	-3.037	0.069962	STK39	1.0655	0.700221
HERC3	-2.6578	0.020277	TIPARP	-1.427	0.342979
HPGD	-2.0116	0.045285	TMPRSS2	-2.0577	0.043524
IGF1R	-2.0479	0.049184	TPD52	-2.6335	0.026346
IGFBP5	1.0841	0.101161	TRIB1	-1.4319	0.444507
IRS2	-1.6835	0.118206	TSC22D1	-2.216	0.044714
JUN	-1.0436	0.926472	TSC22D3	-1.7487	0.114683
KLK2	-2.8097	0.009208	VAPA	-1.6904	0.169269
KLK3	-1.9642	0.129541	VIPR1	-1.6018	0.025484
KLK4	1.1909	0.407188	WIPI1	1.3647	0.273765
KRT8	-1.1503	0.779502	ZBTB10	-1.1935	0.584875
LIFR	-1.5469	0.168466	ZBTB16	-1.5105	0.108269
LRIG1	-1.5578	0.106212	ZNF189	-2.0552	0.071295
LRRFIP2	-2.0359	0.052622	ACTB	-1.5088	0.291685

MAF	-1.1133	0.561656	B2M	1.0743	0.649051
MAP7D1	-1.1021	0.837968	GAPDH	-2.4307	0.023495
MME	-3.3395	0.016146	HPRT1	-2.651	0.005073
MT2A	-2.4456	0.000789	RPLP0	1	0
MYC	-3.6785	0.000886	HGDC	1.0841	0.101161
NCAPD3	-4.1731	0.010971	RTC	1.053	0.649571
NDRG1	1.1548	0.529154	RTC	1.029	0.755263
NFKB1	-1.5773	0.163635	RTC	-1.0034	0.997412
NFKB2	-1.0936	0.86407	PPC	1.1074	0.150436
NFKBIA	-1.2895	0.500925	PPC	1.0029	0.93002
NKX3-1	-2.6587	0.026431	PPC	1.0354	0.385984

Fold changes in the 84 Androgen Receptor Signaling Targets present in the RT[2] profiler PCR array.

Figure S4 (contains full blot of each western)

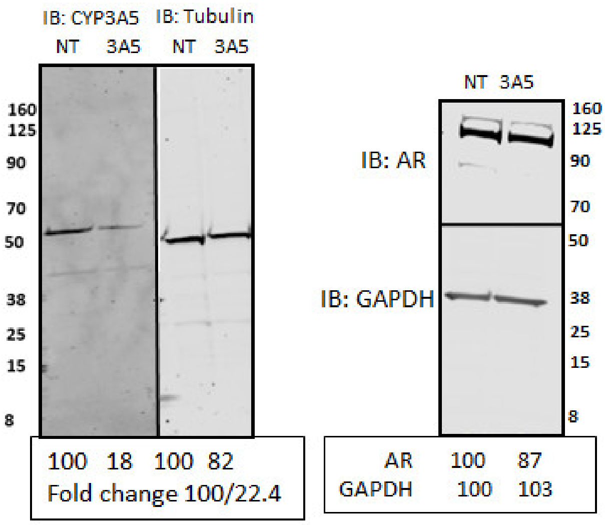

Blots corresponding to Figure 1A

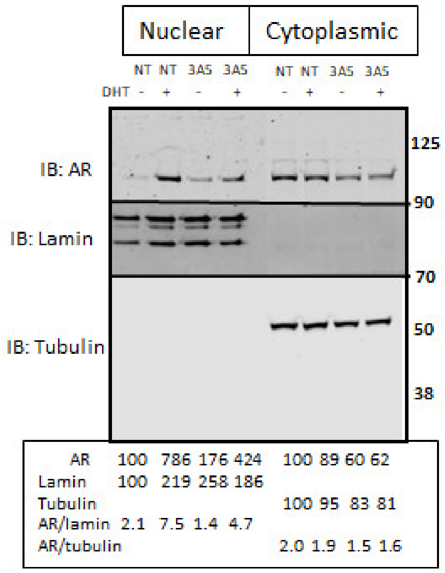

Blot corresponding to Figure 1C

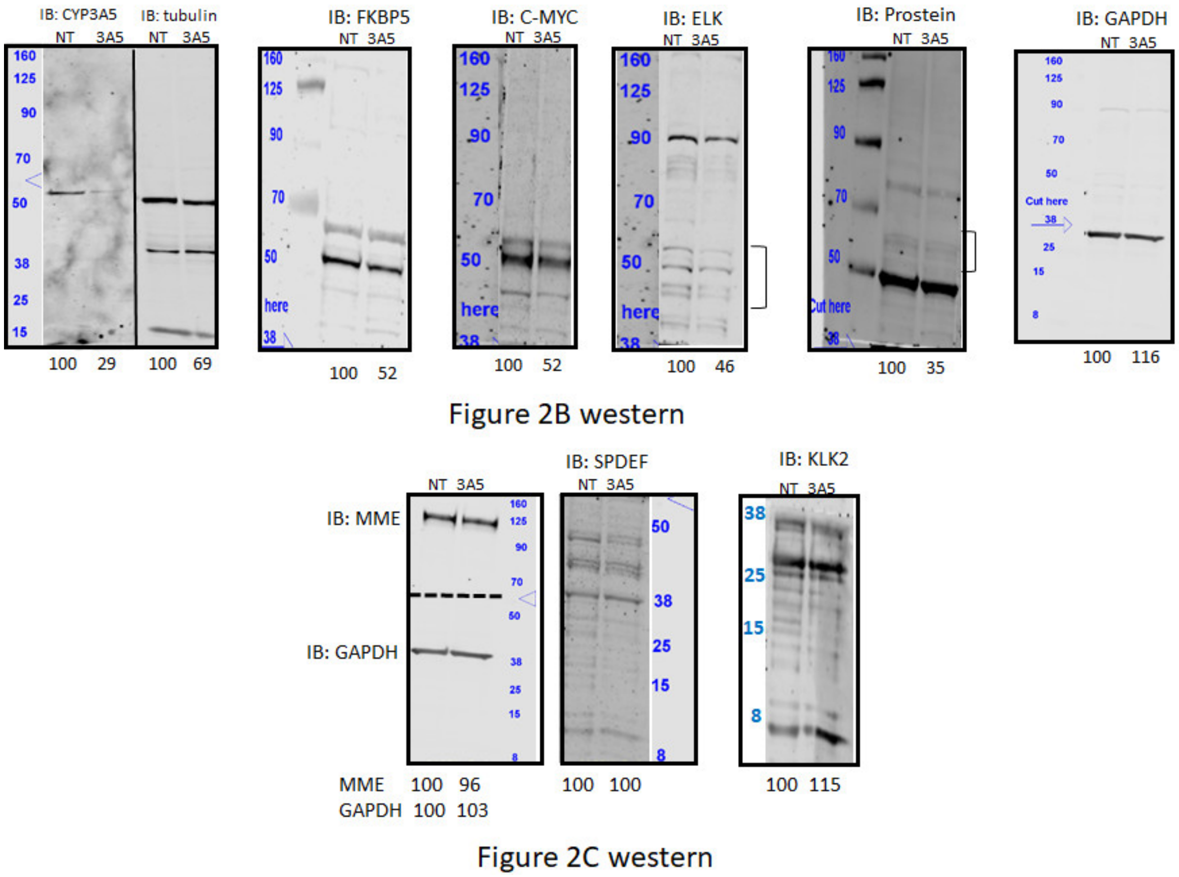

Blot corresponding to Figures 2B and 2C

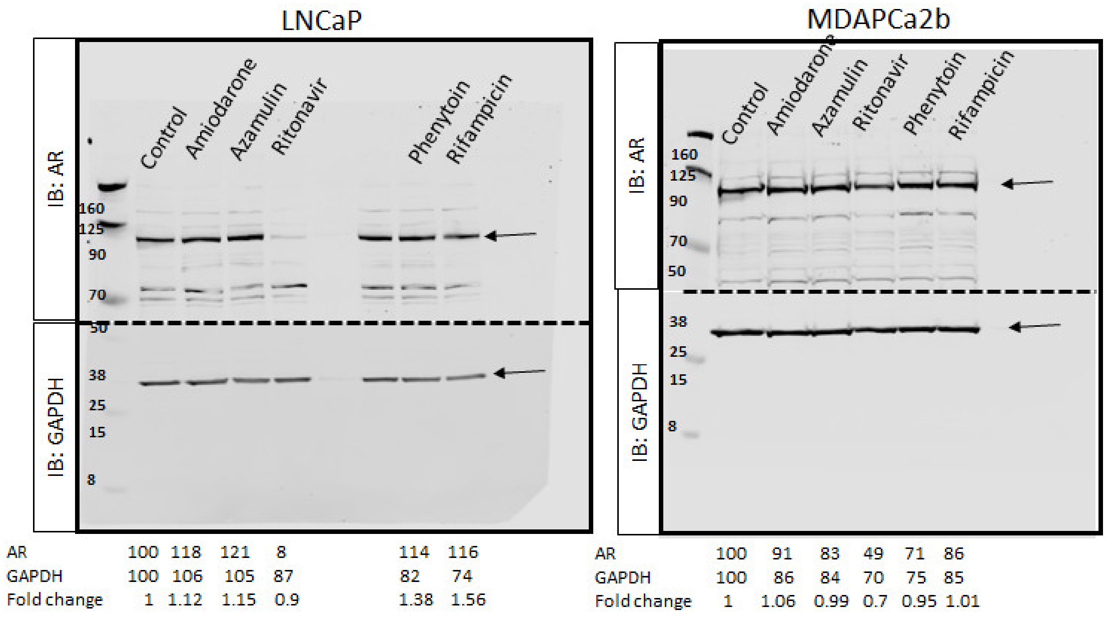

Blots corresponding to Figure 3A

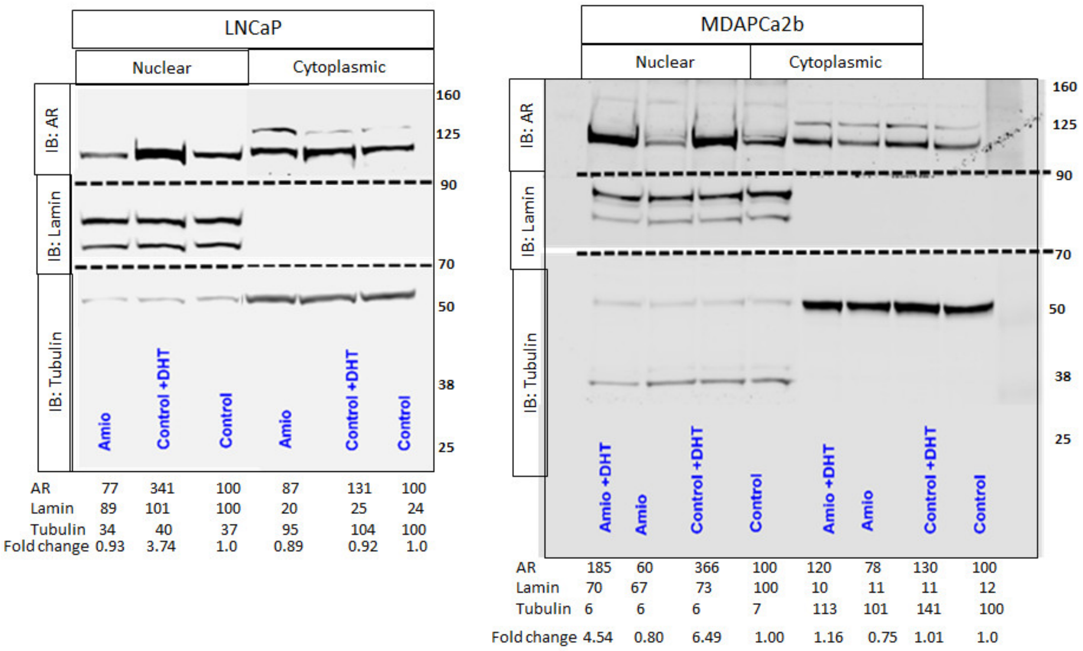

Blots corresponding to figure 3C1

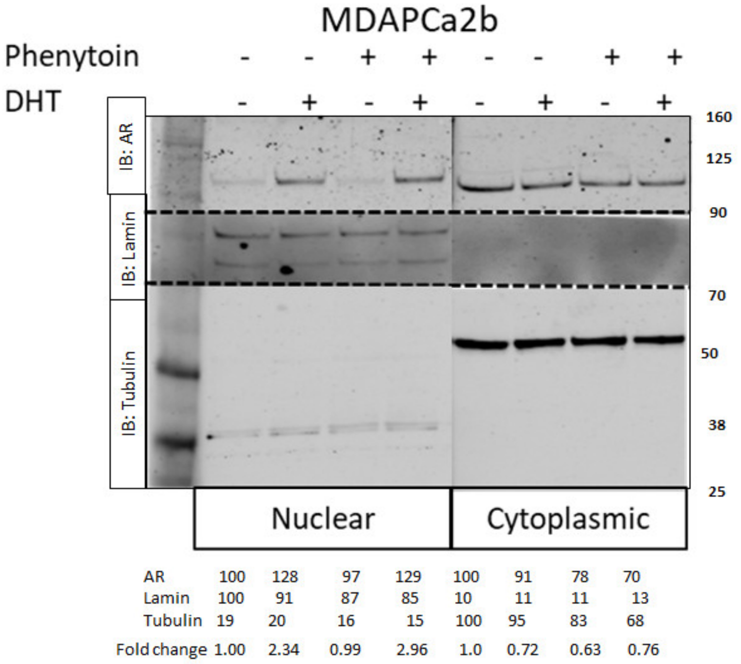

Blot corresponding to Figure 3C2

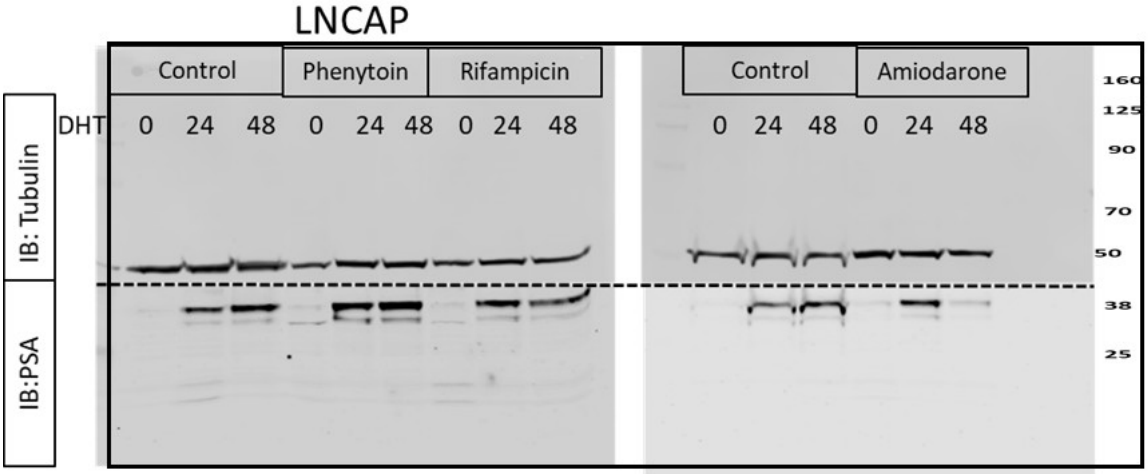

	Control 0	Control 24	Control 48	Phenytoin 0	Phenytoin 24	Phenytoin 48	Rifampicin 0	Rifampicin 24	Rifampicin 48	Control 0	Control 24	Control 48	Amiodarone 0	Amiodarone 24	Amiodarone 48
PSA	100	919	1890	175	2919	3773	165	1750	1699	100	828	1250	107	865	193
Tubulin	100	112	110	58	83	89	64	85	86	100	98	74	87	89	74
Fold change	1	16.36	34.36	4.30	76.66	91.26	2.1	48.27	44.51	1	17.83	36.17	1.09	19.68	4.17

Blot Corresponding to Figure 5A (LNCaP)

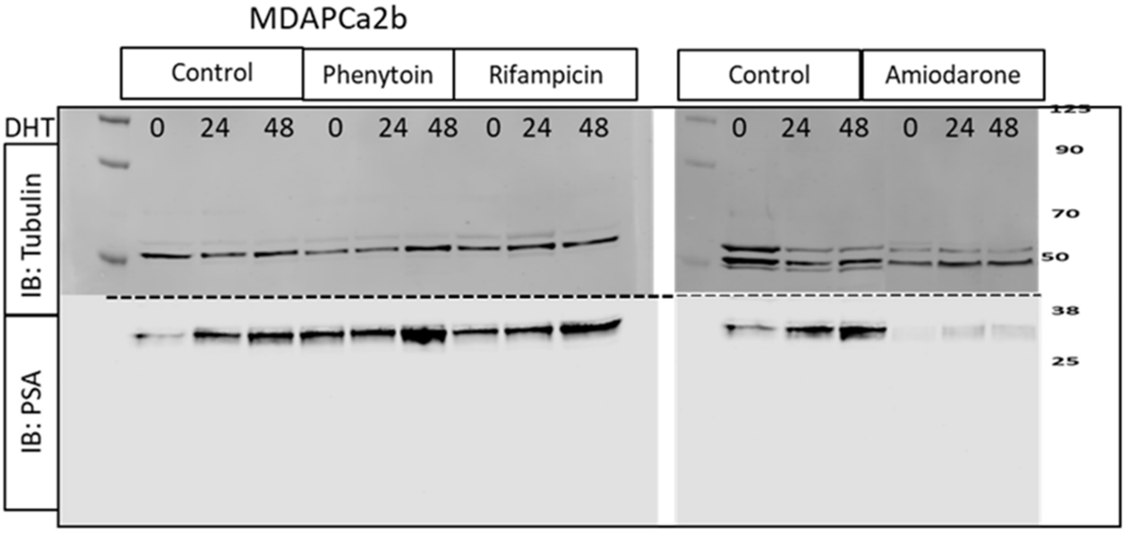

	Control 0	Control 24	Control 48	Phenytoin 0	Phenytoin 24	Phenytoin 48	Rifampicin 0	Rifampicin 24	Rifampicin 48	Control 0	Control 24	Control 48	Amiodarone 0	Amiodarone 24	Amiodarone 48
PSA	100	302	418	380	392	1102	269	391	943	100	217	323	10	32	26
Tubulin	100	88	87	72	58	104	71	104	105	100	109	104	95	95	98
Fold change	1	4.73	5.57	9.06	10.94	11.80	4.88	8.74	12.30	1	1.59	3.88	0.08	0.32	0.25

Blot Corresponding to Figure 5A (MDAPCa2b)

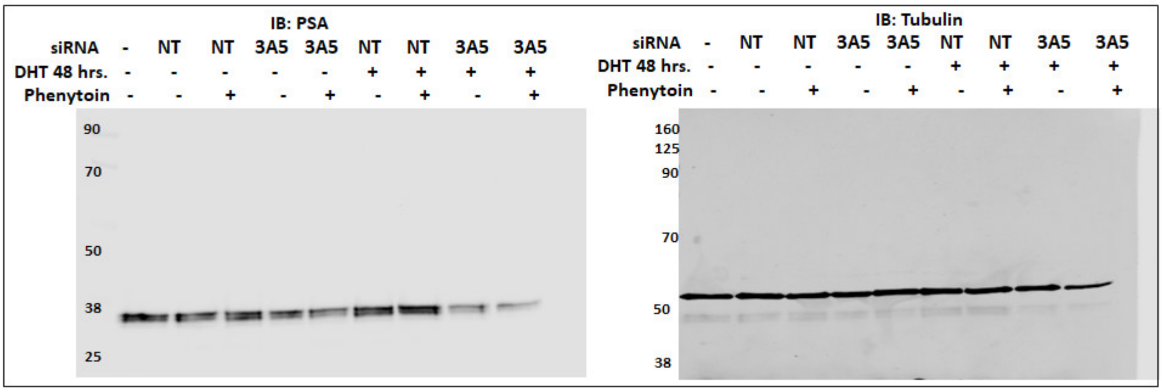

PSA 100 104 73 67 117 137 50 34
Fold change 100 110 91.6 42.9 112.8 195.8 64.7 69.1

Tubulin 100 91 80 153 102 72 76 73

Blots Corresponding to Figure 5B
